# A Comparative Study of The Results of Conventional Surgery and Diode Laser in Maxillary Labial Frenectomy: A Randomized Clinical Trial: Conventional Surgery versus Diode Laser in Maxillary Labial Frenectomy

**DOI:** 10.31661/gmj.v13iSP1.3688

**Published:** 2024-12-31

**Authors:** Hojatollah Yousefimanesh, Parvin Salehi, Elham Maraghi, Dariush Johari

**Affiliations:** ^1^ Department of Periodontics, School of Dentistry, Ahvaz Jundishapur University of Medical Sciences, Ahvaz, Iran; ^2^ Department of Biostatistics and Epidemiology, Faculty of Public Health, Ahvaz Jundishapur University of Medical Sciences, Ahvaz, Iran

**Keywords:** Conventional Surgery, Diode Laser, Maxillary Labial Frenectomy

## Abstract

**Background:**

The frenum, a flexible tissue structure connecting the lips, tongue, and cheeks to the gingiva, can exhibit abnormalities leading to dental complications such as diastema and restricted movement. This study investigates the efficacy and safety of frenectomy by diode laser, compared to traditional surgical approaches.

**Materials and Methods:**

This clinical trial, approved by the ethical committee and registered with the Iranian Registry of Clinical Trials (IRCT20231009059673N1), enrolled 26 patients requiring maxillary labial frenectomy due to papillary or penetrating frenum types who had referred to specialized periodontics department of Jundishapur Dental School of Ahvaz in 2023-2024. Participants were randomly assigned to either a conventional surgical method or the frenectomy by diode laser (Quicklase laser, UK). Surgical procedures were standardized, and various outcome measures—including surgery duration, pain assessment, surgical difficulty, bleeding intensity, swelling, and tissue repair—were evaluated and compared among groups.

**Results:**

This study evaluated the outcomes of maxillary labial frenectomy using laser versus conventional methods in 26 participants (19 women, 7 men). Significant differences were found in surgery duration (laser: 341.46 seconds vs. conventional: 675.00 seconds, P 0.001) and pain levels, with the laser group reporting lower pain on Days One (3.53 vs. 6.00, P 0.001) and Seven (0.46 vs. 1.38, P = 0.002). The laser group also experienced less bleeding and swelling, higher healing scores at one week (3.92 vs. 2.61, P 0.001), and required fewer analgesics (5.53 vs. 9.76, P 0.001), demonstrating superior outcomes with laser treatment.

**Conclusion:**

In conclusion, the England Quicklase laser method demonstrates significant advantages over conventional frenectomy techniques, including shorter surgery duration, reduced pain and bleeding, improved healing, and lower analgesic use. These findings support the laser’s potential as a preferred option for maxillary labial frenectomy, enhancing patient care.

## Introduction

The frenum is a flexible tissue structure composed of mucous membrane and connective tissue fibers, acting as an anatomical connection between the lips, tongue, cheeks, and the underlying periosteum of the gingiva [1]. Its most common attachment sites are located in the labial regions of the maxilla and mandible, particularly around the central incisor and canine-premolar areas [2]. Abnormalities in the size or attachment point of the frenum can lead to a range of dental complications, including diastema, restricted lip movement, and difficulties in speaking and chewing [3]. These issues not only impact oral function but may also contribute to aesthetic concerns and hinder effective oral hygiene practices, potentially leading to plaque accumulation and subsequent oral health problems [4].

From a clinical perspective, the maxillary labial frenum can be classified into four distinct categories based on the orientation of its fibers: mucosal, gingival, papillary, and papillary penetrating. Among these classifications, the papillary and papillary penetrating types are considered pathological due to their association with gingival erosion, diastema formation, loss of papillary tissue, and increased plaque accumulation [5]. A study conducted by Mirko et al. reported that these pathological frenum types have a prevalence of approximately 19% within the population, underscoring the necessity for surgical intervention in certain cases [6].

Frenectomy is the surgical procedure indicated for addressing these abnormalities. This procedure involves a full-thickness incision to completely detach the frenum from its underlying bone before excision [1]. Various surgical techniques are available for performing frenectomy, including conventional scalpel surgery, electrocautery, and laser-assisted methods. The choice of technique often depends on factors such as availability of resources, effectiveness, and patient-specific considerations [7].

In recent years, laser technology has emerged as a popular option in oral surgery since its introduction in the early 1990s [8]. Different types of lasers—including diode, CO2, Nd:YAG, and Er:YAG—are now utilized for soft tissue procedures like frenectomy [9]. Among these options, diode lasers have gained significant traction due to their effective absorption characteristics in water-rich tissues and their advantages in terms of reduced intraoperative bleeding, minimized postoperative pain, shorter recovery times, and overall improved patient comfort [10][11][12].

Despite these advancements, there remains a lack of comprehensive studies comparing diode laser techniques with traditional surgical methods regarding clinical outcomes and complications. Therefore, this study aims to investigate these differences through a clinical trial to provide clearer insights into the efficacy and safety of various frenectomy techniques.

## Materials and Methods

### Study Design

This clinical trial was conducted following the approval of the ethical committee and the acquisition of the Iranian Registry of Clinical Trials (IRCT) code of IRCT20231009059673N1. A total of 26 patients requiring maxillary labial frenectomy due to a papillary frenum or papilla penetrating frenum were enrolled in the study after obtaining informed consent. Inclusion Criteria were as follows: 1. Patients with a papillary type or penetrating papilla frenum, 2. Good systemic health, 3. Good oral hygiene. The exclusion criteria were: 1. Patients with uncontrolled systemic diseases (e.g., diabetes), 2. Poor oral hygiene, 3. Heavy smokers, 4. Immunocompromised individuals, 5. non-cooperative patients.

### Randomization

Participants were randomly assigned to one of two groups using a permuted block randomization method with a block size of 4. The unit of randomization was the individual and the randomization tool was the Rand function in excel. Random sequences were generated by excel, written on cards, sealed in opaque envelopes, and subsequently numbered. During the participants’ enrollment, the envelopes were opened in order, revealing assigned group for each individual. Therefore, nobody knew the assignment until the moment of allocation. Group A: Conventional frenectomy method. And group B: frenectomy by diode

## laser

**Figure-1:**
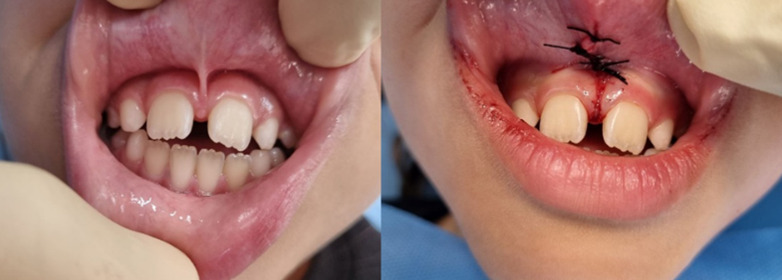
Figure 1. Pre-operative (left) and post-operative (right) images of a patient who underwent treatment using conventional method.

**Figure-2:**
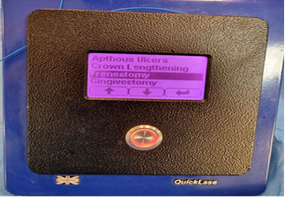
Figure 2. QuickLase Diode laser

**Figure-3:**
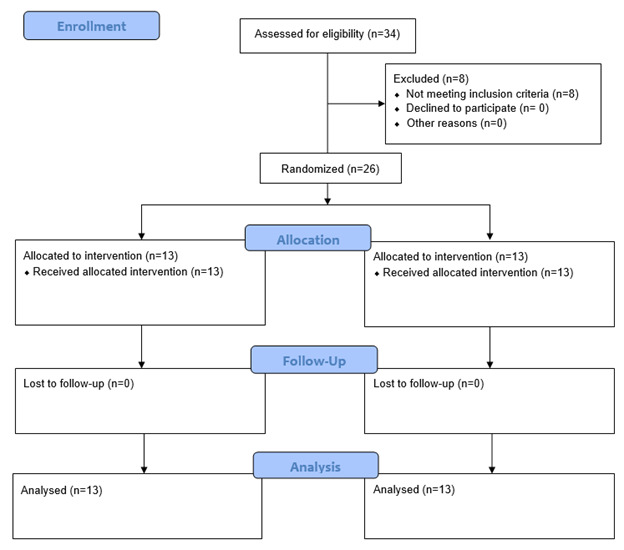
Figure 3. flow diagram of the included participants

### Surgical Procedures

Group A: Conventional Method

Infiltration anesthesia was administered using 2% lidocaine with 1:80,000 epinephrine (Xylopen-Iran). The frenum was grasped using a hemostat. Then a continuous incision was made with a No. 15 blade to separate the frenum from the underlying bone. Interrupted sutures were applied using size 0-4 silk suture thread (Supa-Iran) [7]. Figure-1 illustrates the pre-operative (left) and post-operative (right) images of a patient who underwent treatment using conventional method.

Group B: frenectomy by diode laser

The same anesthetic protocol as Group A was utilized. The England Quicklase diode laser operating at wavelengths of 810-980 nm was employed at a power setting of 2.5 watts in continuous mode (Figure-2). The laser fiber tip was placed in contact with the tissue to separate the frenum from the underlying bone. Remaining tissues were cleaned with sterile gauze soaked in saline. Stitches were not routinely used; however, if bleeding occurred, interrupted sutures with size 0-4 silk suture thread were applied as needed.

### Outcome Measures

The following parameters were assessed for each participant: Surgery Duration, which was recorded in seconds for each procedure [3]. Pain Assessment was evaluated on days 1 and 7 post-surgery using a Visual Analog Scale (VAS) [7]. The Surgical Difficulty was rated by the operating surgeon on a four-point scale [1], with ratings ranging from 1 (very easy) to 4 (impossible). Pain Reliever Usage was documented on day 7 post-surgery [1]. Bleeding Intensity was assessed during surgery and on day 7 post-operation according to WHO criteria [13][14], with a scale from 0 (no bleeding) to 4 (debilitating bleeding). Swelling Assessment was measured on days 1 and 7 post-surgery using a four-point scale [2], where 0 indicated no swelling and 3 indicated severe swelling. Tissue Repair Evaluation was assessed at one week and one-month post-surgery using the Landry Index, rated from 1 (poor) to 5 (excellent) [1].

### Data Collection

All surgeries and data recording were performed by a single operator to ensure consistency across procedures. Follow-ups were conducted 1 week and 1-month post-surgery to monitor recovery and assess long-term outcomes.

### Statistical Analysis

To compare quantitative variables, the independent T-test was utilized when the data distribution was normal. In instances where the data did not conform to a normal distribution, the Mann-Whitney U test was employed. All statistical analyses were conducted using IBM SPSS Statistics software, version 20. A significance level of 0.05 was set for all tests to determine statistical significance.

## Results

**Table:** Table1. Baseline characteristics of study participants

**Variable**	**Conventional Group (n=13) **	**Laser Group (n=13) **	**Total (n=26) **	**P-value**
**Gender**				0.852
Women	8 (61.5%)	11 (84.6%)	19 (73.1%)	
Men	5 (38.5%)	2 (15.4%)	7 (26.9%)	
**Frenum Connection **				0.789
Papillary	9 (69.2%)	9 (69.2%)	18 (69.2%)	
Penetrating the Papilla	4 (30.8%)	4 (30.8%)	8 (30.8%)	
**Educational Background **				0.984
University	7 (53.8%)	7 (53.8%)	14 (53.8%)	
Non-University	6 (46.2%)	6 (46.2%)	12 (46.2%)	

**Table:** Table2. The outcomes of the laser and conventional methods for maxillary labial frenectomy

variables	Conventional (Mean ± SD)	Laser (Mean ± SD)	P-Value
Age (years)	24.53 ± 7.62	25.00 ± 10.95	0.902
Surgery duration (min)	675.00 ± 56.54	341.46 ± 32.75	0.000 ^*^
Pain (day 1) - VAS (cm)	6.00 ± 0.57	3.53 ± 0.96	0.000 ^*^
Pain (day 7) - VAS (cm)	1.38 ± 0.65	0.46 ± 0.66	0.002 ^*^
Complexity (score)	1.92 ± 0.27	1.07 ± 027	0.000 ^*^
Bleeding (day 1) - VAS (cm)	1.00 ± 0.00	0.00 ± 0.00	0.000 ^*^
Bleeding (day 7) - VAS (cm)	0.00 ± 0.00	0.00 ± 0.00	1.000
Swelling (day 1) - VAS (cm)	1.30 ± 0.48	0.61 ± 0.50	0.003 ^*^
Swelling (day 7) - VAS (cm)	0.00 ± 0.00	0.00 ± 0.00	1.000
Healing 1 week (score)	2.61 ± 0.50	3.92 ± 0.27	0.000 ^*^
Healing 1 month (score)	4.84 ± 0.37	5.00 ± 0.00	0.149
Analgesics used (times used in last month)	9.76 ± 1.16	5.53 ± 0.66	0.000 ^*^

^*^Statistically significant

The study included a total of 26 participants (13 in conventional and 13 in laser group) with no lost to follow-up (Figure-3), consisting of 19 women (73.1%) and 7 men (26.9%).

Among these participants, the frenum connection was classified as papillary in 18 individuals (69.2%) and as penetrating the papilla in 8 individuals (30.8%). In terms of educational background, 14 participants had completed university education, while 12 participants had non-university education. No differences were seen in gender, etiology, and educational status of study groups (P > 0.05), as shown in Table-1.

The results of the statistical analysis comparing the outcomes of the laser and conventional methods for maxillary labial frenectomy are presented in Table-2.

The analysis revealed no significant difference in age between the two groups (P = 0.902). However, a significant difference was observed in surgery duration, with the laser group experiencing a shorter duration compared to the conventional group (P < 0.001). Pain levels were significantly lower in the laser group on both Day One (3.53 vs. 6.00, P < 0.001) and Day Seven (0.46 vs. 1.38, P = 0.002). The complexity of the procedure was rated significantly lower in the laser group (P < 0.001).

Bleeding was significantly less in the laser group on Day One, with no bleeding reported in this group compared to a mean score of 1 in the conventional group (P < 0.001). On Day Seven, no bleeding was reported in either group (P = 1.000). Swelling on Day One was also significantly reduced in the laser group (mean score of 0.61 vs 1.30, P = 0.003), with no swelling reported by either group on Day Seven (P = 1.000). Healing scores at one-week post-surgery were significantly higher for the laser group compared to the conventional method (mean score of 3.92 vs 2.61, P < 0.001). However, there was no significant difference in healing scores at one-month post-surgery (P = 0.149). Finally, participants in the laser group required significantly fewer analgesics compared to those in the conventional group (5.53 vs .9 .76, P < 0 .001).

## Discussion

The findings of the present study, which compared the diode laser method to conventional scalpel techniques for maxillary labial frenectomy, align closely with existing literature that supports the efficacy and advantages of laser-assisted procedures in oral surgery. This discussion will compare our results with similar studies to contextualize the benefits observed in our research.

In the present study, the laser technique significantly reduced surgery duration compared to the conventional method (341.46 seconds versus 675.00 seconds). This finding is consistent with previous studies, such as those by Sarmadi et al. [8] Lebret et al. [15] and Xie et al. [3], which reported that laser-assisted frenectomies resulted in shorter surgical times due to the precision and efficiency of laser technology. For instance, Sarmadi et al. found that laser surgery was markedly faster than scalpel surgery, supporting the notion that lasers streamline surgical procedures by minimizing tissue manipulation and coagulation time [8]. These findings align closely with those of the meta-analysis conducted by Prota’sio et al. [12].

Moreover, a systematic review indicated that laser techniques generally lead to shorter operative times and less surgical complexity due to their ability to achieve hemostasis during cutting, which corroborates our findings on reduced complexity ratings for the laser procedure [15]. This efficiency not only benefits the surgeon but also enhances patient experience by reducing time spent in surgery.

Our results showed significantly lower pain levels in the laser group on both Day One and Day Seven post-surgery. This aligns with findings from multiple studies, including those by Yadav et al. which reported that patients undergoing laser frenectomies experienced less pain during and after the procedure compared to those treated with traditional methods [7]. For example, Yadav et al. and Xie et al, noted that patients receiving laser treatment had lower Visual Analog Scale (VAS) scores for pain, indicating a more comfortable experience [3][7].

The reduction in pain can be attributed to the precision of lasers, which minimizes trauma to surrounding tissues and reduces inflammation. As highlighted in a systematic review by Dioguardi et al. lasers provide excellent hemostasis and cause less injury to adjacent tissues, leading to decreased postoperative discomfort [16]. This is particularly relevant in frenectomy procedures where minimizing trauma is crucial for patient satisfaction.

The significant reduction in bleeding observed in our study for the laser group is consistent with other research findings. For instance, Yadav et al. reported significantly less intraoperative bleeding with laser-assisted frenectomy compared to scalpel techniques [7]. Similarly, Lebret et al. noted that laser procedures resulted in lower bleeding rates during surgery, supporting the assertion that lasers provide better hemostatic control [15]. Sobouti et al. also compared 2 different wavelengths (445nm and 980nm) of diode laser and scalpel technique. they observed a significant advantage while using lasers, with the 980nm diode laser having a slightly better control in bleeding [17].

Additionally, our study found less swelling on Day One in the laser group, which aligns with findings from a scoping review that emphasized reduced edema following laser treatments due to their minimally invasive nature [16]. The ability of lasers to coagulate blood vessels while cutting contributes to decreased inflammatory responses and faster recovery times.

Our results indicated significantly better healing scores at one-week post-surgery for the laser group compared to the conventional group. This finding is supported by studies such as those conducted by Sarmadi et al., which showed that patients undergoing laser frenectomies had improved healing outcomes within a short timeframe [8]. Although no significant differences were observed at one month post-surgery in our study, it is essential to note that early healing outcomes can significantly impact overall treatment success and patient satisfaction.

The reduced requirement for analgesics among patients in the laser group further highlights the advantages of this technique. Our findings are consistent with those of previous studies indicating that patients undergoing laser procedures require fewer analgesics due to lower pain levels experienced during recovery [3][7]. This reduction not only enhances patient comfort but also minimizes potential side effects associated with pain medications.

Studies have shown that diode laser frenectomy can result in significantly lower intra and postoperative complications compared to traditional scalpel techniques [18]. Additionally, diode lasers have been found to be effective in reducing pain and promoting wound healing [18][19]. A retrospective study found that diode laser-assisted frenectomy was effective in preventing recurrence of frenulum attachment in patients with abnormal frenulum insertions [20]. Another study compared the use of 445 nm and 980 nm diode lasers versus surgical scalpel and found that diode laser frenectomy resulted in significantly lower intra and postoperative complications [17]. These findings were consistent with current study.

### Limitations and Future Research

Despite these promising results, it is important to acknowledge certain limitations inherent in this study. The relatively small sample size may limit the generalizability of the findings. Additionally, variations in individual patient responses to treatment can influence outcomes; therefore, larger studies with more diverse populations are needed to validate these results further.

Future research should also explore long-term outcomes beyond one-month post-surgery to assess the durability of healing and functional improvements associated with both techniques. Investigating patient-reported outcomes related to quality-of-life post-frenectomy could provide valuable insights into the broader implications of choosing laser versus conventional methods.

## Conclusion

In conclusion, the findings of this study suggest that the diode laser method offers significant advantages over conventional frenectomy techniques, including reduced surgery duration, lower pain levels, decreased bleeding and swelling, improved healing outcomes, and reduced analgesic requirements. These benefits underscore the potential of laser technology as a preferred option for maxillary labial frenectomy procedures, ultimately enhancing patient care and satisfaction in clinical practice. Further research is needed to explore long-term outcomes and broader applications of this innovative surgical approach.

## Conflict of Interest

None.
